# Neurocognitive deficits after botulism: a clinical case series study

**DOI:** 10.3389/fneur.2024.1453653

**Published:** 2024-09-11

**Authors:** Laura Rosenqvist, Charlotte Sandvei, Sigurdur Skarphedinsson

**Affiliations:** ^1^Clinical Center for Emerging and Vector-Borne Infections (CCEVI), The Department of Infectious Diseases, Odense University Hospital (OUH), Odense, Denmark; ^2^Center for Counselling and Vocational Rehabilitation of Brain Injury, Odense, Denmark

**Keywords:** botulism, long-term effects, cognitive impairment, neuropsychological assessment, case series study

## Abstract

**Purpose:**

This case study examined long-term cognitive deficits after botulism. Only a very limited number of studies on post-acute cognitive impairment after botulism exist, and data are incomplete.

**Method:**

A semi-structured interview on long-term cognitive consequences of botulism was conducted for six family members, who contracted the infection after ingestion of lumpfish-roe 2.5 years ago. Two of the family members underwent neuropsychological assessment of attention, memory, and executive functioning as well.

**Finding:**

Results of the semi-structured interviews showed individual subjective cognitive deficits across processing speed, attention, concentration, short-and long-term memory, and executive functioning. Test results showed mild cognitive impairment in attention and mild–moderate deficits in executive functioning.

**Conclusion:**

These results support previous findings that patients of various infectious diseases may suffer unspecific long-term neurocognitive deficits. Assessment and initiation of relevant post-acute treatment and rehabilitation might be central to prognosis, functional ability, and psychological well-being.

## Introduction

Botulism is a rare infectious disease with a reported 0.02 cases per 100,000 inhabitants in EU/EEU countries (1), and annually, 0–2 incidences in Denmark (2). The infection has one of the most potent and severe toxins in nature and is potentially lethal (3).

“Botulism, or “sausage poisoning,” was first described in the early 1800s in a case of ill-preserved blood sausages” (4). The most typical clinical syndromes are foodborne botulism, wound botulism, and infant botulism. All clinical syndromes are broadly characterized by the same neurological symptoms, including blurred or double vision, dry mouth, and difficulty speaking. In severe cases, flaccid paralysis may lead to respiratory distress or failure (3).

Botulism is caused by neurotoxins produced by the bacteria *Clostridium botulinum* and a few other neurotoxic clostridia species. The neurotoxin blocks the release of acetylcholine from the presynaptic nerve terminal in neuromuscular junctions, causing bilateral flaccid paralysis or muscle weakness. Recent evidence indicates that botulism not only affects peripheral nerve endings but may also impact the central nervous system (CNS) by retrograde transport of the toxin as well as indirectly through neural plasticity [for review of studies, see (5)]. Experimental studies on rodents have shown that botulinum toxin can indeed penetrate the blood–brain barrier [for review of studies, see (6)].

In light of evidence that botulism affects CNS (5), neuropsychological sequelae of the disease may be expected, but empirical evidence on the long-term neurocognitive effects of botulism is very limited. Two case studies, both examining foodborne botulism, have described broad neurocognitive implications and memory-specific effects of botulism (30, 7).

A case study on general neuropsychological effects after botulism (30) concluded that there were no deficits of higher cognitive functioning in the patient group (*N* = 4) immediately after acute treatment of the infection. However, one patient did in fact show mild cognitive impairment on tests on concept formation, learning and recall, and mental flexibility. Furthermore, two other patients did not participate in several tests due to a weakened condition and a lack of cooperation.

Another case study on botulism and memory performance (7) found no significant differences in learning, immediate verbal and nonverbal memory, storage, and retrieval between botulism patients (*n* = 8) and a matched control group of patients (*n* = 8) 1 year after onset of symptoms. However, the patient control group was not defined in terms of disease, and test performances were not compared to performances of a healthy control group or validated norms.

### The present study

Overall, neuropsychological pioneer studies have examined neurocognitive effects after botulism and concluded no clear cognitive impairments (30, 7), but current clinical findings in a group of botulism patients at Odense University Hospital show opposing symptoms. To the best of our knowledge, no studies have examined long-term effects of botulism across neurocognitive domains. Furthermore, central methodological limitations of the previous case studies warrant a revision of the implications of botulism on CNS.

In the present paper, we aim to study potential long-term neurocognitive effects of botulism by conducting a clinical case series study through clinical interviews and neuropsychological assessments.

## Methods

### Participants

The design was reviewed by the Regional Committees on Health Research Ethics for Southern Denmark who found no ethical-scientific reasons against this study (case number S-2023200-4). The case study consists of data from six family members contaminated with *Clostridium botulinum* from lumpfish roe in March 2021. We refer to Table 1 for the demographic and clinical characteristics of the case subjects. All participants delivered their informed consent to participate in the present study.

**Table 1:** Descriptive statistics of the clinical case subjects (*N* = 6).

	Age	Occupation	Symptom onset (days after infection)	Length of hospitalization (days)	Treatment	Charlson Comorbidity Index score
Daughter	46	Administrative employee and teacher	8	2	None	0
Daughter’s husband	49	Self-employed	2	10	Botulinum antitoxin Nasogastric tube Pyridostigmine	1
Mother	71	Retired, former sales person	9	0	None	3*
Father	74	Retired, former business-owner	7	1	Pyridostigmine	3*
Aunt	74	Retired, former bank employee	2	8	Botulinum antitoxin Pyridostigmine	3*
Uncle	81	Retired, former business manager	2	5	Botulinum antitoxin Pyridostigmine	6*

*Respectively 3 and 4 points (Uncle) are given due to age only.

### Materials

#### Semi-structured interview

The neuropsychological assessment consisted firstly of a semi-structured interview about the premorbid medical and psychiatric history, the course of the botulism infection, subjectively experienced effects of medical treatment and rehabilitation, self-reported physical symptoms and cognitive difficulties post-infection, current and previous psychological reactions to the infection, coping, and daily and occupational functioning compared to the pre-infection level. Furthermore, the participants stated their educational and occupational history to allow estimation of premorbid cognitive capability and level of functioning. Regarding present symptoms, the time frame was defined as the previous 4 weeks. The mean duration of each interview was approximately 1 h 15 min.

A Charlson Comorbidity Index score (8) was calculated for each family member in order to take potential complexity of comorbidity into account. Five family members received a score higher than 0, three of them due to age only and not specific medical conditions.

#### Neuropsychological test battery

The test battery consisted of 13 tests of processing speed, attention, memory, and executive functioning. Tests on processing speed were conducted at the beginning and end of the test battery to assess possible fatigue effects on cognitive performance. In between memory tasks, tests with the least possible interference with recall in memory were conducted. We refer to Table 2 for a conceptual overview of each test.

**Table 2:** Description of the neuropsychological test battery.

Name of test	Central cognitive domain
Trail Making Test A (15)	Processing speed, executive functioning
Trailmaking Test B (15)	Executive functioning
RAVLT (Rey Auditory Verbal Learning Test) (16)	Auditory memory
RCFT (Rey Complex Figure Test and Recognition Trial) (17, 18)	Visuospatial memory
Digit Span Forward (19)	Attention
Digit Span Backward (19)	Attention, working memory
Matrix Reasoning (19)	Perceptual reasoning, visuospatial ability
Coding (19)	Processing speed
Vocabulary (19)	Verbal comprehension
Verbal fluency – semantic (20)	Executive functioning, vocabulary knowledge
Verbal fluency – phonetic (20)	Executive functioning, vocabulary knowledge
The Five-Point Test (21)	Executive functioning
Color-Word Interference Test (20)	Executive functioning
The D2 Test of Attention (22, 23)	Selective attention, concentration, processing speed

The order of the tests is: (1) Trailmaking Test A, (2) Trail Making Test B, (3) RAVLT (Learning Trial, Distraction list, and Short recall), (4) RCFT (Copy Trial), (4) Digit Span Forward, (5) Digit Span Backwards, (6) RCFT (Immediate recall), (7) Matrix Reasoning, (8) Coding, (9) RAVLT (Late recall and Recognition), (10) RCFT (Recognition Trial), (11) Vocabulary, (12) Verbal fluency, (13) The Five-Point Test, (14) Color-Word Interference Test, and (15) The D2 Test of Attention. Central cognitive domains descriptions are inspired by Groth-Marnat and Wright (24).

### Procedure

Two individuals (Daughter and Daughter’s husband) were referred to the Clinic of Infectious Diseases at Odense University Hospital in August 2023 due to post-infectious symptoms after botulism. Due to complaints of persevering cognitive deficits, a neuropsychological assessment was conducted by the first author of the present paper (an authorized clinical psychologist) in August and September 2023. The purpose of the neuropsychological assessment was to inform possible rehabilitation and recommendations on the course of treatment.

The assessment consisted of a semi-structured interview and neuropsychological testing. The total duration of each session was 3 h 10 min for the Daughter and 3 h for the Daughter’s husband. For the purposes of the present paper, the remaining four family members were invited to contact the clinic if they wished to inform us about potential long-term cognitive consequences of botulism. All four family members reached out within a week. They were informed that the purpose of the study was to gather preliminary data on long-term cognitive deficits after a botulism infection. All family members approved the use of their anonymized clinical information in the present study. Semi-structured interviews similar to the interviews of the first two patients were administered via telephone. Assessments and interviews were all conducted between 29 and 30.5 months post-infection.

## Results

We refer to Table 3 for an overview of somatic and cognitive symptoms of all six case subjects.

**Table 3:** Overview of immediate and long-term symptoms of the family members.

	Daughter	Daughter’s husband	Mother	Father	Aunt	Uncle
Symptoms at the onset of infection						
Blurred or double vision	X	X	X	X	X	X
Dizziness and balance problems	–	X	X	X	–	–
Fatigue	X	X	X	–	X	–
Breathlessness	–	–	–	–	X	X
Word-finding problems, speech impairments	–	X	–	–	X	X
Paralysis	–	X	–	–	–	–
Reduced sense of smell and hearing	–	–	X	–	–	–
Unspecific bodily pain	–	–	–	–	–	X
Reduced muscular strength	X	X	–	–	–	–
Urinary incontinence	–	–	–	–	–	X
Persevering somatic and cognitive symptoms						
Fatigue	X	X	X	–	–	–
Sensitivity to visual and auditory stimuli, tinnitus	X	X	–	–	–	–
Sensory disturbance	–	X	–	–	–	–
Headache and migraine	X	X	–	–	–	–
Shaking, including tremor	X	X	–	–	–	
Dizziness and balance problems	–	–	X	X	–	–
Reduced sense of smell and hearing	–	–	X	–	–	–
Reduced mental processing speed	X	X	–	X	–	X
Attention problems	X	X	X	X	-	X
Memory problems	X	X	–	–	X	X
Word-finding problems	X	–	–	–	X	X
Executive functioning problems	–	X	X	X	X	X
Emotional regulation problems	X	X	X	X	X	X
Impaired everyday functioning	X	X	X	X	–	–

### Results of semi-structured interviews

#### Somatic symptoms

All family members reported blurred or double vision shortly after the infection. Other symptoms included paralysis in the neck region, dizziness and balance problems, reduced sense of smell and hearing, breathing difficulty, loss of energy, non-specific bodily pain, reduced muscular strength, and urinary incontinence. Two family members experienced a clear decline in the above symptoms after 1 year. Two family members reported substantial symptom remission after 3 weeks and 1.5–2 years, respectively. The two youngest family members still describe substantial persisting symptoms with the development of new somatic symptoms during the aftermath of the infection such as tremors and migraine.

Some somatic symptoms have persisted until the present day, 2.5 years after the time of infection. These include fatigue, dizziness and balance problems, reduced sense of smell and hearing, sensitivity to auditory and visual stimuli, tinnitus, sensory disturbances, frequent headaches and migraine, and tremor.

#### Cognitive symptoms

All family members were confident that the cognitive symptoms below had developed after the infection. They reported that the cognitive problems had been present from the start of the infection, although the symptoms were given less attention the first months due to the acute somatic symptoms. As a part of the interview, the family members were asked to differentiate between cognitive problems generated from fatigue and cognitive problems independent from energy levels. We included only the latter in the results in order to describe subjective cognitive difficulties as valid as possible. Four family members described the long-term cognitive difficulties as stable, while the two youngest family members have experienced a worsening since the onset of symptoms. None of the family members have received medical treatment for their cognitive symptoms nor cognitive rehabilitation.

Four family members reported present slow mental processing (e.g., substantially increased latency when processing new information). Five family members described attention difficulties (e.g., shifting attention between daily tasks, reading, and staying focused in everyday conversations). Three family members described problems with long-term memory (e.g., remembering names and recent events, as well as an elongated retrieval time for episodic memories), three family members had difficulties with short-term memory as well (e.g., forgetting items when grocery shopping), and two family members described specific memory encoding and storing difficulties (e.g., forgetting the plot in a book). Three family members reported word-finding difficulties. Five family members reported reduced executive functioning (e.g., problems in multitasking between chores, and lower mental flexibility when plans change). Furthermore, five family members described changes in emotional reactions (e.g., greater irritability and overall lower threshold for emotional reactions).

All family members denied core symptoms of depression, anxiety, and post-traumatic stress-disorder in line with clinical impressions and medical journals.

#### Everyday functioning

While the four oldest family members were already retired, the two youngest family members were business owners at the time of infection. Today, both are unable to work. Four family members experience limitations in their everyday functioning compared to levels of functioning before infection. The persevering symptoms have substantially reduced the frequencies and durations of social engagement, voluntary work, household chores, creative projects, and gardening.

### Results of neuropsychological testing

Table 4 shows neuropsychological test descriptives for the Daughter and Daughter’s husband. In both subjects, processing speed decreased from the first to the last test, indicating possible fatigue effects. This corresponds with both patients’ reporting on VAS (Visual Analogue Scale) before and after testing, on which both patients reported mental exhaustion after testing.

**Table 4:** Neuropsychological test results for the *n* = 2 patients.

	Daughter		Daughter’s husband	
	Raw score	Percentile	Raw score	Percentile
Processing speed				
Coding	81	91	65	50
Attention				
Digit Span Forward	7	16	10	75
Digit Span Backward	9	63	6	16
D2-test – Total score	431	42	434	42
D2-test – Error (%)	1.86	75–90	2.07	75–90
D2-test – Concentration performance	172	66	171	62
Memory				
RCFT – Copy	31	8	31	8
RCFT – Time	229	38	118	86
RCFT – Immediate recall	16	18	25.5	79
RCFT – Recognition	21	53	23	85
RAVLT – Learning trials	69	99	64	97
RAVLT – Immediate recall	15	95	14	91
RAVLT – Late recall	15	95	14	91
RAVLT – Recognition	15/15	N/A	15/15	N/A
Perceptual reasoning				
Matrix reasoning	18	50	12	16
Verbal comprehension				
Vocabulary	38	50	43	63
Executive functioning				
Trailmaking Test A	27	64	22	79
Trailmaking Test B	75	44	63	51
Verbal Fluency Test – Semantic	22	29	21	24
Verbal Fluency Test – Phonetic	18	64	7	5
The Five-Point Test (perseveration error)	27 (1)	30	48 (5)	96 (10)
Color-Word Interference – Color naming (error)	33 (1)	25 (18)	37	9
Color-Word Interference – Word reading	25	25	32	2
Color-Word Interference – Inhibition (error)	66 (3)	16 (5)	54	50
Color-Word Interference – Inhibition/switching (error)	61 (1)	50 (20)	60 (1)	50 (63)

An normal range performance is defined as percentile 16–84. Percentile 2–16 translates to a mildly reduced performance, percentile 0.1–2 is moderately reduced, and less than percentile 0.1 is severely reduced (25). Fractional percentiles are rounded off to the nearest integer. Interpretation of test results is based on Scandinavian norms for WAIS-IV (26) and D-KEFS (27), German norms for D2-test (23), American norms for RCFT (17), and Danish norms for the remaining tests (28, 29).

Auditory attention span was below the expected performance, considering age and premorbid functioning. A fluctuating working memory performance indicated attention span difficulties as well.

There were executive difficulties across tests. Both patients showed mild-to-moderate reductions in visuo-constructive organization. An increased error rate in figural fluency indicated difficulties in monitoring one’s own mental processes. Executive difficulties with verbal fluency and inhibition indicated auditory-based executive problems as well.

## Discussion

The present case study aimed to examine long-term cognitive deficits after botulism. The main finding was that all six family members reported subjective cognitive impairments more than 2.5 years post-infection. The cognitive reductions were not confined to a single cognitive domain but appeared to affect processing speed, attention span and shifts, memory, and executive functioning. Neuropsychological testing indicated mild-to-moderate cognitive impairments concerning attention and executive functioning. Furthermore, a declining processing speed indicated increasing effects of fatigue during testing sessions.

The present case study is the first empirical description of the long-term effects of botulism across cognitive domains. Our findings appear to support previous findings that patients of various infectious diseases may suffer unspecific long-term neurocognitive deficits. For example, a subgroup of patients of COVID-19 (9) and neuroborreliosis (10) show unspecific neurocognitive deficits post-infection. As such, long-term neurocognitive impairments seem to appear across certain viral infections, bacterial infections with no toxin, and toxin-mediated bacterial infections. Choutka et al. (11) have recently suggested that this pattern of post-infectious syndromes across infections could be explained by a unifying pathophysiology. The microbiological mechanisms, however, remain hypothetical at this point.

In addition, our findings align with recent empirical evidence indicating that the effects of botulism neurotoxins are not limited to the peripheral nervous system but may, in fact, imply CNS [see (5)]. We conclude this based on our observations of unspecific cognitive reductions coinciding with the onset of the infection. While all six family members reported subjective long-term cognitive impairments after the infection, two of the family members indicated signs of deficits in the neuropsychological testing as well. The noticeable differences in acute somatic symptoms between the two family members who underwent neuropsychological assessment and the comparable test results suggest that neither onset of symptoms, symptom severity, nor length of hospital stay are strong confounders of long-term cognitive impairment. The amount of ingested botulinum toxin does not appear to be convincingly associated with cognitive impairments either. We know that the quantity of ingested botulinum toxin is related to onset of symptoms and severity of the botulism infection (3), and while the amount of toxin each family member ingested is unknown, there was a tendency in our patient group that relatively early onset of symptoms (i.e., 2 days after ingestion) was associated with longer stays at the hospital (i.e., 5–10 days), whereas family members with relatively late onset (i.e., 7–9 days after ingestion) had no or relatively short stays (i.e., 0–2 days). However, there was no clear associations across family members between onset of symptoms and extent and severity of cognitive impairment. It could be the case that other mechanisms than toxin quantity are associated with the long-term neurocognitive consequences of a botulism infection. Future studies aiming to determine underlying mechanisms of long-term cognitive impairments should ideally recruit a larger sample of patients.

In the following, we discuss the differences between the results of the present case study and the two previous case studies on the impact on neurocognition after botulism.

Concerning botulism and memory, Haaland and Davis (7) detected no immediate impairments on Corsi’s nonverbal span and supraspan tests and Buschke’s Selective Reminding Test, and no impairments were evident on a serial testing of Hebb’s verbal test up to 1 year post-infection. Test results were not compared to norms or the test results of a healthy control group which makes comparison problematic.Despite methodological challenges, however, our results did also not indicate hippocampal damage or focal neurologic deficits. In contrast to Haaland and Davis’ (7) patients, who denied experiencing memory problems, our patients did report memory-specific problems in the semi-structured interviews. Considering their general test performances, however, the experience of reduced memory could likely be related to a limited attention span and executive functioning problems, which were evident from the neuropsychological testing. An impaired encoding process might explain the experienced difficulties associated with retrieval, recall, and recognition.

Comparisons with the study of Graebner and Cleeland (30) pose problems due to incomplete data in their report (e.g., omitted test results due to patient condition and lack of cooperation). However, in one out of two complete data sets, Graebner and Cleeland’s (30) patient did report mild cognitive impairments of concept formation, visuospatial recall, and mental flexibility skills. The mild reduction of test performance across cognitive domains corresponds overall with our test results. Graebner and Cleeland estimate that the remaining three test performances are within the normal range despite the results being partial. Of course, we cannot reject that some botulism patients do not show long-term neurocognitive deficits post-infection. It is indeed promising if a group of patients show a better outcome than our patients. More research is needed on mapping the long-term neurocognitive effects after botulism to better understand the expected trajectories.

Lastly, it is central to consider the relationship between neurocognitive impairments and fatigue. Fatigue occurs as a residual symptom after botulism (12), and our patients did report symptoms of fatigue. Since correlations between fatigue and cognitive functioning have been found in some studies [however, inconsistently, (13)], it is central to consider whether neurocognitive impairments can be attributed to low energy. Notably, however, the clinical subjects in the present study reported cognitive difficulties independently of energy levels, and two family members denied fatigue symptoms and still described cognitive impairments. Based on self-report, this may suggest that long-term neurocognitive difficulties after botulism and possibly other infections constitute a phenomenon of its own. Furthermore, attributing neurocognitive impairments after botulism solely to fatigue may be reductionistic, since fatigue is already prevalent in the general healthy population (11, 14). Attributing cognitive sequelae only to fatigue may thus risk overlooking potential underlying mechanisms in CNS of long-term cognitive impairments after various infections.

### Limitations

We point to two possible limitations of the present study.

First, as four out of six family members were above 70 years old, cognitive impairments could be attributed to other etiologies than botulism. To evaluate this, the patients were assessed during hospitalization for other etiologies of cognitive deficits. These assessments did not cause the patients’ medical practitioners to pursue different diagnostic explanations in the subsequent years after the time of infection. In addition, the family members themselves were confident that the cognitive deficits had developed distinctively after the infection.

Second, the present case study relies partly on self-report with a risk of bias, as negativity bias and groupthink may influence reporting. The family members may have described their symptoms as worse than they actually were and are due to the negative valence of the experience. Furthermore, the narrative may have homogenized in the family since the family members have relied on each other for confidence and support. However, each patient delivered individual examples of symptoms and interference with everyday life, and as such, the symptoms clearly appeared to have individual character.

### Directions for research and treatment

Due to the rarity of (human) botulism cases, generalizability is limited. In order to qualify neuropsychological prognosis after post-acute treatment of botulism, studies with increased sample sizes are needed. Considering the low number of annual cases, this may demand a transnational study.

Clinically, it appears to be highly relevant to offer neuropsychological assessment to initiate relevant post-acute treatment, e.g., cognitive remediation therapy, occupational therapy, and psychotherapy. In line with our patients’ reports, cognitive impairment may be overshadowed by acute somatic symptoms early in the course of the disease with a risk of overlooking treatable difficulties with long-term implications. These long-term consequences of botulism may include loss of vocational ability and potentially quality of life. The need for long-term treatment and rehabilitation likely vary across individuals, underlining the importance of individual neuropsychological assessments.

## Data Availability

The raw data supporting the conclusions of this article will be made available by the authors, without undue reservation.
